# Notes from the University of Pennsylvania Veterinary Department

**Published:** 1898-04

**Authors:** 


### NOTES FROM THE UNIVERSITY OF PENNSYLVANIA VETERINARY DEPARTMENT.

The examinations in the special course in horseshoeing will be completed early in April.

A dog-ambulance has been added to the equipment of the hospital.

The capacity of the hospital for small animals has been enlarged by the addition of six cages of special design, which can be used for either cats or small dogs.

The early graduates of the Veterinary Department were surprised and gratified to see the changes and improvements that have been made in the school and surroundings during the past year.

Some very instructive investigations in relation to the milk of tuberculous cows are now under way, the milk being derived from three fresh cows in the tuberculous-cattle section of the hospital.

The clinics have been larger during the past year, and a greater number and variety of cases have been presented for examination, demonstration, and treatment, than ever before in the history of the Veterinary Hospital.

The regular Monday afternoon "field-work" for last week consisted in a visit to the Pasteurizing plant and milk-depot of the largest milkdealer in Philadelphia, Mr. George Abbott. Next week the senior class will visit some dairy and breeding-farms in Chester County.

During the past year ten of the recent graduates of the school have received official veterinary appointments.

It appears that a new avenue for veterinary work is opening—there is a growing demand for veterinarians to act as managers of stock- and dairy-farms.

The new course of lectures on veterinary jurisprudence, ethics, and business methods, by Dr. W. Horace Hoskins, has developed as everyone expected, and has been most interesting and instructive. College instruction on these subjects has never, heretofore, been a prominent feature, in America at least—perhaps on account of the difficulty of obtaining a lecturer competent to handle the matter; but the success and value of the present course will probably make it a permanent part of the curriculum.

In a few months the street in front of the buildings will have been so improved that the whole aspect of the institution will be changed. The city has ceded Pine Street from Thirty-sixth to Thirty-ninth to the University, and it is being rapidly transformed from a poorly paved city street into an attractive parkway.

Following the very interesting lecture on pigeons by Mr. Atwood B. Hoskins, the Veterinary Society was fortunate in obtaining the services of Mr. Thomas Sharpless to lecture on swine, with especial reference to Chester Whites. Mr. Sharpless's long and successful experience as a breeder, and his careful study of the problems of this art, together with his high culture and facility of expression, make a combination of rare qualifications.

---

CONCERNING the results of Dieckerhoff's treatment for azoturia, District Veterinarian Schmidt gave five horses with azoturia one hundred grammes of bicarbonate of soda every four hours. Three horses were cured in three days, one in five days, and the other was killed on account of extensive injuries.

The New York State Veterinary College has received one hundred and seventy-five thousand dollars from the State Treasury.

